# Ischemic Stroke and Dietary Vitamin B12 Deficiency in Old-Aged Females: Impaired Motor Function, Increased Ischemic Damage Size, and Changed Metabolite Profiles in Brain and Cecum Tissue

**DOI:** 10.3390/nu14142960

**Published:** 2022-07-19

**Authors:** Joshua Poole, Paniz Jasbi, Agnes S. Pascual, Sean North, Neha Kwatra, Volkmar Weissig, Haiwei Gu, Teodoro Bottiglieri, Nafisa M. Jadavji

**Affiliations:** 1 College of Osteopathic Medicine, Midwestern University, Glendale, AZ 85308, USA; joshua.poole@midwestern.edu (J.P.); snorth45@midwestern.edu (S.N.); 2 Biomedical Sciences Program, College of Graduate Studies, Midwestern University, Glendale, AZ 85308, USA; apascu@midwestern.edu (A.S.P.); nkwatra46@midwestern.edu (N.K.); vweiss@midwestern.edu (V.W.); 3 College of Health Solutions, Arizona State University, Phoenix, AZ 85281, USA; pjasbi@asu.edu (P.J.); hgu@fiu.edu (H.G.); 4 School of Molecular Sciences, Arizona State University, Tempe, AZ 85308, USA; 5 College of Dental Medicine Arizona, Midwestern University, Glendale, AZ 85308, USA; 6 Department of Pharmaceutical Sciences, College of Graduate Students, Midwestern University, Glendale, AZ 85308, USA; 7 Department of Environmental Health Sciences, The Robert Stempel College of Public Health and Social Work, Florida International University, Miami, FL 33199, USA; 8 Center for Translational Science, Cellular Biology and Pharmacology Department, The Herbert Wertheim College of Medicine, Florida International University, Port St. Lucie, FL 33199, USA; 9 Center of Metabolomics, Institute of Metabolic Disease, Baylor Scott & White Research Institute, Dallas, TX 75204, USA; teodoro.bottiglieri@bswhealth.org; 10 College of Veterinary Medicine, Midwestern University, Glendale, AZ 85308, USA; 11 Department of Neuroscience, Carleton University, Ottawa, ON K1S 5B6, Canada

**Keywords:** ischemic stroke, vitamin B12, female, motor function, aging

## Abstract

A vitamin B12 deficiency (vit. B12 def.) is common in the elderly, because of changes in metabolism. Clinical studies have reported that a vit. B12 def. results in worse outcome after stroke, and the mechanisms through which a vit. B12 def. changes the brain requires further investigation. This study investigated the role of vit. B12 def. on stroke outcome and mechanisms using aged female mice. Eighteen-month-old females were put on a control or vit. B12 def. diet for 4 weeks, after which an ischemic stroke was induced in the sensorimotor cortex. After damage, motor function was measured, the animals were euthanized, and tissues were collected for analysis. Vit. B12 def. animals had increased levels of total homocysteine in plasma and liver, and choline levels were also increased in the liver. Vit. B12 def. animals had larger damage volume in brain tissue and more apoptosis. The cecum tissue pathway analysis showed dysfunction in B12 transport. The analysis of mitochondrial metabolomics in brain tissue showed reduced levels of metabolites involved in the TCA cycle in vit. B12 def. animals. Motor function after stroke was impaired in vit. B12 def. animals. A dietary vit. B12 def. impairs motor function through increased apoptosis and changes in mitochondrial metabolism in brain tissue.

## 1. Introduction 

Stroke is among the leading causes of death globally and its prevalence as a major health concern is predicted to increase as the global population ages and the demographics of populations change [1,2]. Currently, stroke is prevalent and detrimental to elderly populations (>65 years old) [1,3]. Ischemic stroke is the most common form of stroke. It is caused by blockage of arterioles leading to portions of the brain. The blockage results in reduced oxygen and energy supply to the brain, causing severe disability and death. Many factors contribute to stroke risk and outcomes, making it highly multifactorial. Nutrition is a modifiable risk factor for stroke [2,4]. For example, a vitamin B12 deficiency is a well-established risk factor for stroke and worse stroke outcomes. Approximately 20% of older adults (>60 years old) have a vitamin B12 deficiency, making it of high concern to this population [5,6]. 

Cardiovascular disease (CVD) is the leading cause of death among men and women. Women have more risk factors and worse outcomes than men with CVD [7] One of the many reasons these problems exist is that preclinical studies are targeted towards males [8]. Over 90% of preclinical studies use strictly male mice, whereas all clinical studies use equal numbers of male and female participants [8,9]. This makes clinical pharmaceutical findings favor better outcomes in males [10,11]. This approach is taken despite stroke frequency and outcomes in female mice and human participants changing depending on age, menopause, and other female-specific biological factors that are not applicable in males. Assessing these differences and strengthening female treatment is lost in the lack of female focus in preclinical studies [8,11]. Another aim of this paper is to bridge this gap and increase insight into the female-specific stroke mechanisms and treatment. 

Vitamin B12 is a component of one-carbon (1C) metabolism, which is a network that integrates nutritional signals with biosynthesis, redox homeostasis, and epigenetics, and plays an essential role in the regulation of cell proliferation, stress resistance, and embryo development [8]. A vitamin B12 deficiency results in increased levels of homocysteine, which is a risk factor for stroke. The literature demonstrates that patients with a vitamin B12 deficiency and hyperhomocysteinemia during an ischemic stroke have been reported to have worse outcomes [5,12,13,14]. Vitamin B12 plays an essential role in mitochondrial energy production and cellular function [15]. Mitochondria are a major contributor to the development of apoptotic and necrotic cell death after ischemic stroke [16]. The impact of vitamin B12 deficiency after stroke on mitochondrial function requires further investigation. The literature shows a clear link between a dietary deficiency in vitamin B12 and an increased risk for ischemic stroke and worse outcome, but the mechanisms remain unknown. Stroke outcomes in women are not well understood [7]. This study adds an understanding of the mechanisms through which a dietary vitamin B12 deficiency changes the female brain and behavior using a mouse model system. 

## 2. Materials and Methods

### 2.1. Animals 

All experiments in animals were approved by the Midwestern University IACUC committee. Female C57BL/6J mice were obtained from Jackson Laboratory for this study. Twenty-two 18-month-old female mice were obtained and, upon arrival, were habituated for 1 week prior to the start of the experiments. 

### 2.2. Experimental Design 

**Figure 1:**
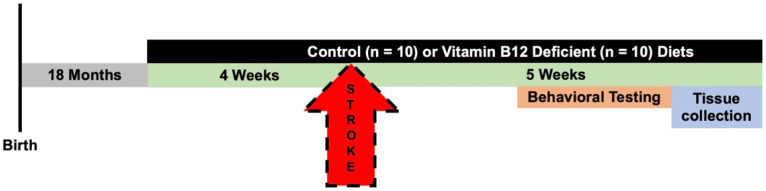
Timeline of experimental manipulation. Female C57Bl/6J mice arrived from Jackson Laboratories at 10 months old, and after one week of acclimation, the animals were placed on a control (*n* = 10) or vitamin B12-deficient (*n* = 10) diet for four weeks. Following the four weeks, ischemic stroke was induced using the photothrombosis model in the sensorimotor cortex. After stroke, the animals were maintained on their respective diets for four additional weeks. The motor function of the mice was measured using the accelerating rotarod and forepaw placement tasks. At the completion of the in vivo experiments, the animals were euthanized, and brain and liver tissue, as well as plasma, was collected for further analysis.

An overview of all experimental manipulations is outlined in Figure 1. After the mice were habituated to the Midwestern University animal facility, the mice were randomly assigned to control (*n* = 11) or vitamin B12-deficient (*n* = 11) groups and placed on diets for 4 weeks. At 18 months of age, ischemic stroke was induced in the animals using the photothrombosis model [17,18,19,20,21]; this corresponds to middle-age in humans [22]. The ischemic stroke damaged the sensorimotor cortex, which allows for motor function assessment. Four weeks after damage, motor function was measured in the animals using the forepaw placement, accelerating rotarod, and ladder beam tasks. After the completion of behavioral testing, the animals were euthanized and tissue was collected, including brain, liver, cecum, and blood tissue, for further study.

### 2.3. Diet

The mice were placed on a vitamin B12-deficient (vit. B12 def.) (0 mg/kg, *n* = 10) or a control (0.025 mg/kg vitamin B12, *n* = 10) diet four weeks prior to photothrombosis damage and four weeks post-photothrombosis damage. The diets were formulated by and purchased from Envigo. The control diet (TD. 190790) contained the recommended dietary amount of nutrients for mice [23]. The vitamin B12-deficient diet (TD. 190791) was formulated based on a previous study in mice that has shown it to be safe and have no negative side effects [24]. The mice had ad libitum access to food and water throughout the experiment. The body weights of each animal were recorded weekly.

### 2.4. Photothrombosis Model 

Using the photothrombosis model of ischemic stroke damage, the mice were anesthetized with 4–5% isoflurane in oxygen. After anesthetization, the mice had the top of their heads shaved and disinfected. Tear gel was used to prevent their eyes from drying out while anesthetized and 0.03 mg/kg of buprenorphine and 1 mL of saline were administered subcutaneously. The mice were then transferred to a stereotaxic apparatus (Harvard Apparatus) and maintained at 2–2.5% isoflurane. The mice were placed on a heating pad and a probe was rectally inserted to maintain a body temperature of 37 °C. Prior to laser exposure, the mice were intraperitoneally injected with 10 mg/kg of photoactive Rose Bengal (Sigma, Burlington, MA, USA) followed by a 5 min delay to allow the dye to enter circulation. The skin at the top of the head was surgically cut to expose the skull and then the sensorimotor cortex was targeted using stereotaxic coordinates (3 cm above, mediolateral + 0.24 mm from the bregma). The skulls of the mice were exposed to a laser (Beta Electronics, St Miami, FL, USA, wavelength: 532 nm) for 15 min. For recovery of post-operative pain, buprenorphine was administered to all animals prior to ischemic damage.

### 2.5. Behavioral Testing 

#### 2.5.1. Bederson Scale and Neurological Scoring Scale

Following stroke, animals subsequently exhibit a variety of neurological deficits. The Bederson scale is a global neurological assessment that was developed to measure neurological impairments following stroke [25]. The tests include forelimb flexion, resistance to lateral push, and circling behavior. A severity scale of 0–3 is used to assess behavioral deficits after stroke. This scoring scale is a simple way to reveal basic neurological deficits. Ischemic animals will have significantly more neurological deficits than non-ischemic animals, resulting in a higher score [25]. 

#### 2.5.2. Accelerating Rotarod 

A standard accelerating rotarod apparatus (Harvard Apparatus) was used to measure walking movements and balance as previously described [19,26,27]. Thirty centimeters above the ground, the mice were placed on a rotating rod 3 cm in diameter and 6 cm wide in which the speed gradually increased from 4 to 60 RPM over 8 min. When the mice fall off the rotarod, a digital sensor records the latency in seconds. An average of three trials per mouse were taken with an intertrial interval of 5 min.

#### 2.5.3. Forepaw Placement 

To measure spontaneous forepaw usage, the mice were placed in a 19 cm high, 14 cm diameter cylinder, and the placement of their forepaws on the cylinder wall during natural exploratory rearing behaviors was recorded using a digital camera for frame-by-frame analysis [19,28]. During a rear, the first forepaw placement on the wall was recorded as impaired, non-impaired, or both. 

#### 2.5.4. Ladder Beam

The ladder rung apparatus was composed of two Plexiglas walls. Each wall contained holes located at the bottom edge of the wall; the holes could be filled with metal bars. The entire apparatus was placed atop two standard mouse housing cages. The performance was video-recorded from the side, with the camera positioned at a slight ventral angle so that all four limbs could be recorded at the same time [29].

All video recordings were analyzed frame-by-frame. Each step was scored according to the quality of the limb placement as previously described [29]. For the analysis of foot placement accuracy, the number of errors in each session was counted. The error score was calculated from the total number of errors and the number of steps for each limb [29].

### 2.6. Total Homocysteine and Choline Metabolite Measurements 

At the time of euthanization, blood was collected by cardiac puncture in EDTA-coated tubes and centrifuged at 7000× *g* for 7 min at 4 °C to obtain the plasma. Liver tissue was also removed at the same time and the samples were stored at −80 °C until the time of the analysis. Total homocysteine (tHcy), *S*-adenosylmethionine, S-adenosylhomocysteine, methionine, cystathionine, betaine, and choline were measured in the plasma and liver by liquid chromatography–tandem mass spectrometry (LC-MS/MS) as previously described [30].

### 2.7. Brain Tissue Processing 

Some mice were perfused, and fixed brain tissue was sectioned using a cryostat at 30 μm and the slides mounted in serial order. There were six slides full of brain tissue sections of the damaged area per mouse, and each animal had a minimum of four sections that were used for quantification. ImageJ (NIH) was used to quantify the ischemic damage volume by measuring the area of damaged tissue [31].

### 2.8. Immunofluorescence Experiments 

Brain tissue was used in immunofluorescence analysis to assess apoptosis, using active caspase-3 (1:100, Cell Signaling Technologies, Danvers, MA, USA, catalog number: 9662). All brain sections were stained with a marker for neuronal nuclei, NeuN (1:200, AbCam, Hong Kong, China, catalog number: ab 104224). Primary antibodies were diluted in 0.5% Triton X and incubated with brain tissue overnight at 4 °C. The next day, the brain sections were incubated in Alexa Fluor 488 (Cell Signaling Technologies, catalog number: 4408) or 555 (Cell Signaling Technologies, catalog number: 4417) and secondary antibodies were then incubated at room temperature for 2 h and stained with 4′,6-diamidino-2-phenylindole (DAPI) (1:1000, Thermo Fisher Scientific, Waltham, MA, USA). The stains were analyzed using a microscope (Zeiss, Oberkochen, Germany) and all images were collected at the magnification of 400×. 

In the brain tissue within the ischemic region, the co-localizations of active caspase-3 with NeuN-labeled neurons were counted and averaged per animal using the Axio Imager (Carl Zeiss, Oberkochen, Germany). A positive cell was indicated by co-localization of the antibodies of interest located within a defined cell. The cells were distinguished from debris by identifying a clear cell shape and intact nuclei (indicated by DAPI and NeuN) under a microscope. All cell counts were conducted by two individuals blinded to the treatment groups. The number of positive cells were counted in three brain sections per animal. For each section, three fields were analyzed. The number of positive cells were averaged for each animal. 

### 2.9. Metabolomics

Some mice from each group were euthanized using a CO_2_ overdose (control diet, *n* = 5; vitamin B12-deficient diet, *n* = 5). Cecum and brain tissue (contralateral and ipsilateral cortices) were micro-dissected and immediately frozen for analysis. Prior to LC-MS/MS-targeted measurement, frozen tissue supernatant samples were first thawed overnight at 4 °C. Afterward, 20 mg of each thawed sample was placed in a 2 mL Eppendorf vial. The initial step for protein precipitation and metabolite extraction was performed by adding 500 μL MeOH and 50 μL internal standard solution (containing 1810.5 μM ^13^C_3_-lactate and 142 μM ^13^C_5_-glutamic acid). The mixture was then vortexed for 10 s and stored at −20 °C for 30 min, followed by centrifugation at 14,000 RPM for 10 min at 4 °C. The supernatants (450 μL) were collected into new Eppendorf vials and dried using a CentriVap Concentrator (Labconco, Kansas City, MO, USA). The dried samples were reconstituted in 150 μL of 40% PBS/60% ACN and centrifuged again at 14,000 RPM at 4 °C for 10 min. Afterward, 100 μL of supernatant was collected from each sample into an LC autosampler vial for subsequent analysis. A pooled sample, which was a mixture of all experimental samples, was used as the quality control (QC) sample and injected once every 10 experimental samples.

The targeted LC-MS/MS method used here was modeled on the approach developed and used in a growing number of studies [32,33,34,35,36]. Briefly, all LC-MS/MS experiments were performed on an Agilent 1290 UPLC-6490 QQQ-MS system. Each supernatant sample was injected twice: 10 µL for analysis using negative ionization mode, and 4 µL for analysis using positive ionization mode. Both chromatographic separations were performed in hydrophilic interaction chromatography mode on a Waters XBridge BEH Amide column (150 × 2.1 mm, 2.5 µm particle size, Waters Corporation, Milford, MA, USA). The flow rate was 0.3 mL/min, the auto-sampler temperature was kept at 4 °C, and the column compartment was set to 40 °C. The mobile phase was composed of Solvents A (10 mM NH_4_OAc, 10 mM NH_4_OH in 95% H_2_O/5% ACN) and B (10 mM NH_4_OAc, 10 mM NH_4_OH in 95% ACN/5% H_2_O). After an initial 1 min isocratic elution of 90% B, the percentage of Solvent B decreased to 40% at t = 11 min. The composition of Solvent B was maintained at 40% for 4 min (t = 15 min), after which the percentage of B gradually went back to 90%, to prepare for the next injection. The mass spectrometer was equipped with an electrospray ionization (ESI) source. Targeted data acquisition was performed in multiple-reaction-monitoring (MRM) mode. The whole LC-MS system was controlled by Agilent MassHunter Workstation software. The extracted MRM peaks were integrated using Agilent MassHunter Quantitative 11.0 Data Analysis software (Agilent, Santa Clara, CA, USA). 

### 2.10. Data Analysis and Statistics

The behavioral and brain tissue data were analyzed by two individuals that were blinded to the experimental treatment groups. GraphPad Prism 6.0 was used to analyze behavioral testing, plasma tHcy measurements, lesion volume, immunofluorescence staining, and choline measurements. In GraphPad Prism 6.0, two-way ANOVA analysis was performed when comparing the mean measurement of both sex and dietary group for behavioral testing, one-carbon metabolites, lesion volume, and immunofluorescence staining. Significant main effects of the two-way ANOVAs were followed up with Tukey’s post hoc test to adjust for multiple comparisons. MetaboAnalyst 5.0 was used to analyze the brain and cecum metabolomics data [37]. The measured metabolites were mapped to the Kyoto Encyclopedia of Genes and Genomes (KEGG) reference database for *Mus musculus* to assess pathway enrichment. The metabolomics data were normalized to tissue weight and log-transformed prior to both univariate and multivariate comparisons. All data are presented as mean ± standard error of the mean (SEM). All statistical tests were performed using a significance level of 0.05. 

## 3. Results

### 3.1. Increased Levels of Homocysteine in Plasma and Liver and Changes in Other One-Carbon Metabolites in Vitamin B12-Deficient Diet Animals

The homocysteine levels were measured in plasma and liver tissues 5 weeks after ischemic stroke. The females maintained on a vit. B12 def. diet had higher levels of plasma (*p* = 0.008) and liver (*p* = 0.0012) total homocysteine levels compared to the controls (Table 1).

There were no differences in other one-carbon metabolites in liver tissue except higher levels of choline (*p* = 0.02) in the vitamin B12-deficient animals compared to the control diet animals (Table 2).

### 3.2. Increased Ischemic Damage Volume in Vitamin B12-Deficient Animals after Stroke

**Figure 2:**
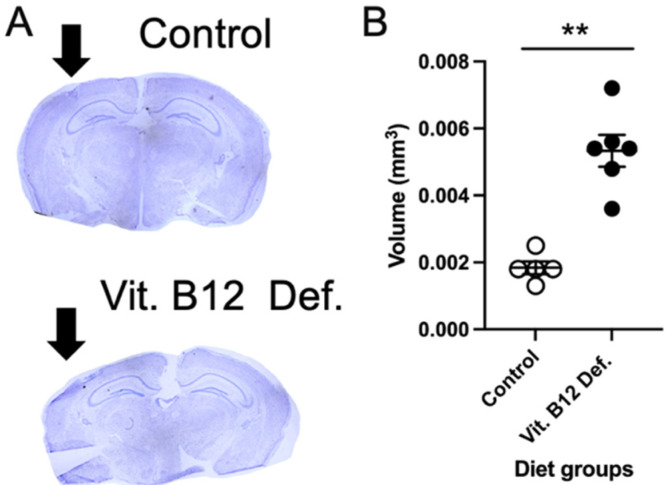
Impact of dietary vitamin B12 deficiency and ischemic stroke on damage volume. Representative cresyl violet image (**A**) and ischemic damage volume quantification (**B**). Depicted are means of ±SEM of four to five mice per group. ** *p* < 0.01, un-paired *t*-test.

The ischemic damage volume was measured in brain tissue 5 weeks following photothrombosis damage. Representative images of cresyl violet-stained brain tissue from the control and vitamin B12-deficient animals are shown in Figure 2A. The quantification of ischemic damage shows that the vit. B12 def. animals had greater damage volume compared to the controls (Figure 2B; *p* = 0.01).

### 3.3. More Neuronal Apoptosis in Vitamin B12-Deficient Animals after Ischemic Stroke

**Figure 3:**
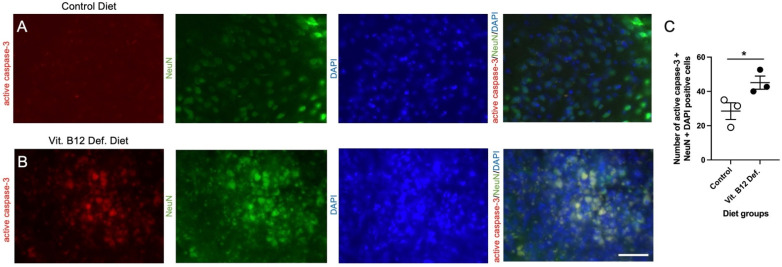
Impact of dietary vitamin B12 deficiency (vit. B12 def.) and ischemic stroke on neuronal active caspase-3 cell counts. Representative images of immunofluorescence staining with positive semi-quantitative spatial colocalization of active caspase-3 with neuronal nuclei (NeuN) and 4′,6-diamidino-2-phenylindole (DAPI) from control (**A**) and vit. B12 def. (**B**) diets. Quantification of active capsapse-3, NeuN, and DAPI cell counts (**C**). Depicted are means of ± SEM of three mice per group. Scale bar = 50 μm. * *p* < 0.05, un-paired *t*-test.

Within the ischemic damage region, we measured levels of neuronal apoptosis using active caspase-3 and NeuN. In Figure 3, representative images of immunofluorescence staining for control (Figure 3A) and vit. B12 def. (Figure 3B) diet animals are shown. The quantification of apoptosis shows that the vit. B12 def. animals had more apoptosis compared to the controls (Figure 3C, *p* = 0.05).

### 3.4. Metabolomic Measurements

#### 3.4.1. Reductions in TCA Cycle in Brain Tissue of Vitamin B12-Deficient Females after Ischemic Stroke

**Figure 4:**
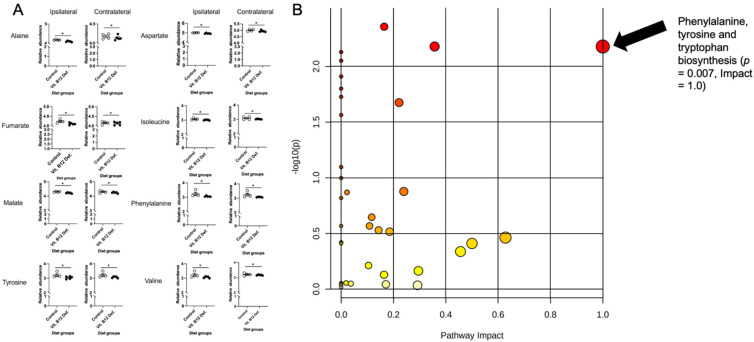
Impact of dietary vitamin B12 deficiency (vit. B12 def.) and ischemic stroke on mitochondria metabolomics in ipsilateral and contralateral brain tissue. Significance testing and enrichment analysis of metabolite data. Data were normalized to sample weight and log-transformed prior to analysis. (**A**) Significant between diet group metabolites (regardless of ischemic damage) as determined by two-way MANOVA: alanine (*p* = 0.027), aspartate (*p* = 0.019), fumarate (*p* = 0.047), isoleucine (*p* = 0.039), malate (*p* = 0.013), phenylalanine (*p* = 0.012), tyrosine (*p* = 0.014), valine (*p* = 0.030). (**B**) Pathway enrichment analysis performed using KEGG canonical pathways. Circle size denotes impact, whereas color corresponds to significance. Enriched pathways with *p* < 0.05 and impact > 0.50 have been labeled along with their p-values and pathway impact scores. * *p* < 0.05, un-paired *t*-test.

In total, 34 compounds were reliably detected in the samples (i.e., QC coefficient of variation (CV) < 20%). A two-factor heatmap showing the normalized relative abundance of captured metabolites by group and lesion status is visualized in Supplementary Figure S1. Pearson’s correlation and clustering analysis revealed mainly positive correlations, with few negative correlations identified (Supplementary Figure S2). Specifically, strong positive correlations (*r* ≥ 0.5) were found within metabolites of phenylalanine metabolism, branched-chain amino acid (BCAA) metabolism, and metabolites of the TCA cycle. Meanwhile, strong negative correlations (*r* ≤ −0.5) were observed between amino acids and TCA-cycle metabolites. An orthogonal partial least squares–discriminant analysis (OPLS-DA) was performed using the total set of 34 detected metabolites to assess brain mitochondria metabolite profiles (Supplementary Figure S3A), and permutation testing with 100 iterations was performed to assess the model fit (Supplementary Figure S3B). The OPLS-DA scores plot showed notable separation between the control and vitamin B12-deficient groups, and the model showed good predictive capacity (*Q*^2^ = 0.435) and high explanatory capacity (*R*^2^ = 0.815). Importantly, permutation testing showed that the model did not overfit the data (perm. *p* < 0.05). A two-way MANOVA with all measured metabolites was performed to test the main effects of ipsilateral and contralateral to ischemic stroke, as well as B12 deficiency on cortical tissue. Although no significant main effect by stroke type was observed (i.e., ipsilateral vs. contralateral to stroke), a significant main effect of vit. B12 def. was observed in eight captured metabolites: phenylalanine (*p* = 0.012), malate (*p* = 0.013), tyrosine (*p* = 0.014), aspartate (*p* = 0.019), alanine (*p* = 0.027), valine (*p* = 0.029), isoleucine (*p* = 0.039), and fumarate (*p* = 0.047). Normalized box plots of significant metabolites are provided in Figure 4A. Unsupervised principal component analysis (PCA) was performed with the subset of eight significant metabolites and showed appreciable separation between the control and vitamin B12-deficient animals, with more than 83% of the total variance explained (Supplementary Figure S4). Furthermore, receiver operating characteristic (ROC) analysis was performed with the eight significant metabolites; we noted that two significant metabolites (phenylalanine and tyrosine) had univariate area under curve (AUC) estimates > 0.90 (Supplementary Figure S5), suggesting these two metabolites to be highly accurate indicators of B12 deficiency. Phenylalanine showed an AUC = 0.988, (95% CI: 0.9–1.0, sensitivity = 0.9, specificity = 1.0), while tyrosine showed a similarly high AUC = 0.925 (95% CI: 0.75–1.0, sensitivity = 0.8, specificity = 0.9).

Additionally, the measured metabolites were annotated using the *Mus musculus* KEGG reference database, and pathway enrichment analysis was performed between the B12-deficient and control animals using a global test of relative distance betweenness centrality. The results are visualized as a scatter plot in Figure 4B, showing significance by pathway impact. One pathway was shown to be significantly impacted between groups: phenylalanine, tyrosine, and tryptophan biosynthesis (*p* = 0.007). Importantly, all metabolites in this pathway were measured by the metabolomic assay, and two showed significance (impact = 1.0). 

**Figure 5:**
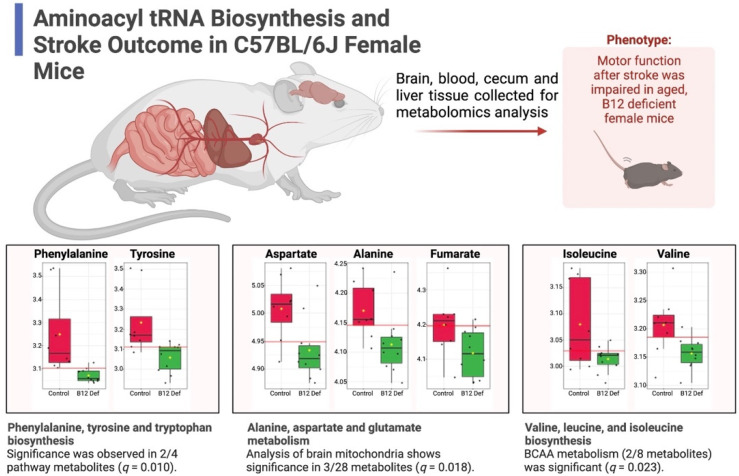
Pathway analysis results summarized in the context of aminoacyl tRNA biosynthesis. Metabolomic analysis (scatter plots) of brain mitochondria suggests involvement of phenylalanine, tyrosine, and tryptophan biosynthesis, alanine, aspartate and glutamate metabolism, and valine, leucine, and isoleucine biosynthesis in impaired motor function following stroke (all metabolites *p* < 0.05). Predicted functional significance (*q*) adjusted for FDR. BCAA, branched-chain amino acids.

The results of our pathway analysis showed significant alterations (FDR *q* < 0.05) in nine canonical murine pathways (Figure 4B): (1) alanine, aspartate, and glutamate metabolism, (2) glycine, serine, and threonine metabolism, (3) cysteine and methionine metabolism, (4) valine, leucine, and isoleucine biosynthesis, (5) lysine biosynthesis, (6) arginine and proline metabolism, (7) histidine metabolism, (8) phenylalanine, tyrosine, and tryptophan biosynthesis, and (9) tryptophan metabolism. Notably, these nine pathways are constitutive of the aminoacyl tRNA biosynthesis superpathway. As such, we have summarized our findings across the subpathways of greatest impact with regard to aminoacyl tRNA biosynthesis in Figure 5. As can be seen, the metabolomic analysis of brain mitochondria implicates dysregulated aminoacyl tRNA biosynthesis as a primary driver of impaired motor function following stroke, particularly in constituent pathways containing phenylalanine, tyrosine, aspartate, alanine, fumarate, isoleucine, and valine. Importantly, these effects are observed at both the metabolite (0.01 ≤ *p* ≤ 0.05) and pathway (0.01 ≤ *q* ≤ 0.02) levels.

#### 3.4.2. Reduced Levels of Creatine and Increased Levels of Methylmalonic Acid in Cecum of Vitamin B12-Deficient Animals after Ischemic Stroke

**Figure 6:**
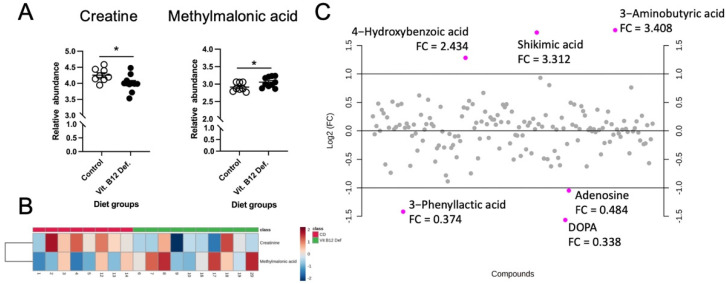
Impact of dietary vitamin B12 deficiency and ischemic stroke on cecum metabolomics. Significance testing, heatmap visualization, and fold change (FC) analysis of metabolite data. Data were normalized to sample weight and log-transformed prior to analysis; samples were unpaired, equal group variances assumed, and parametric test performed. (**A**) Significant between-group metabolites as determined by independent samples *t*-test: creatinine (*p* = 0.04), methylmalonic acid (*p* = 0.04). (**B**) Heatmap display of creatinine and methylmalonic acid. (**C**) FC analysis (performed as B12-deficient/control diet) showed three metabolites with FC > 2 (4-hydroxybenzoic acid, shikimic acid, 3−aminobutyric acid) and three metabolites with FC < 0.5 (3−phenyllactic acid, DOPA, adenosine). * *p* < 0.05, un-paired *t*-test.

In the cecum samples, 158 metabolites were reliably detected after filtering (i.e., QC CV < 20%). The independent samples *t*-testing of metabolites between the vitamin B12-deficient and control diet groups revealed two significant metabolites: creatinine (*p* = 0.04) and methylmalonic acid (*p* = 0.04). Normalized box plots of these significant metabolites are provided in Figure 6A, and are further visualized as a heatmap between the groups in Figure 6B. Fold change (FC) analysis (B12-deficient/control diet) was also performed to assess the magnitude of the changes in metabolites. Although non-significant, three metabolites (3-aminobutyric acid, shikimic acid, and 4-hydroxybenzoic acid) showed FC > 2 and three metabolites (DOPA, 3-phenyllactic acid, and DOPA) showed FC < 0.5. These results are summarized and displayed in Figure 6C. 

### 3.5. Reduced Stroke Outcome in Vitamin B12-Deficient Animals

#### 3.5.1. Higher Neuro Deficit Score in Vitamin B12-Deficient Animals after Ischemic Stroke

**Figure 7:**
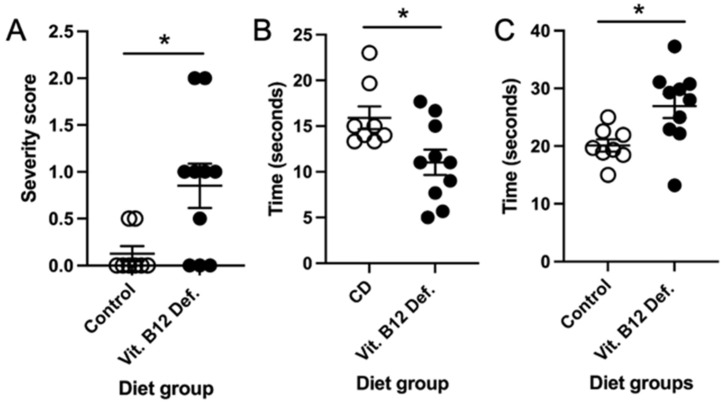
Impact of vitamin B12 deficiency on motor function after ischemic stroke. Neuro deficit score, vertical screen test severity score (**A**). Latency to fall off the accelerating rotarod (**B**). Amount of time to cross the ladder beam (**C**). Eight to ten mice per group. * *p* < 0.05, unpaired *t*-test.

Four weeks after damage, we measured the neuro deficit in the animals. The vit. B12 def animals had a higher severity score on the vertical screen test compared to the control diet animals (Figure 7A, *p* = 0.02). There was no difference in the postural reflex (*p* = 0.50) and forelimb placing (*p* = 0.60) tests.

#### 3.5.2. Impaired Balance and Coordination in Vitamin B12-Deficient Animals after Ischemic Stroke

After ischemic stroke, the vit. B12 def. female mice were not able to stay on the accelerating rotarod as long as the control animals (Figure 7B, *p* = 0.021). There was no difference in the revolutions per minute between the groups (*p* = 0.90). 

#### 3.5.3. No Difference in Forepaw Placement between Dietary Groups

We measured whether there was a difference between forepaw usage after stroke. No difference between vit. B12. def. and controls were observed in impaired (*p* = 0.22) and non-impaired (*p* = 0.098) forepaw usage. 

#### 3.5.4. No Difference in Skilled Motor Function between Dietary Groups

There was no difference in the movement score of the impaired (*p* = 0.72) and non-impaired (*p* = 0.66) limbs. The number of errors made while crossing the ladder did not differ between the control and vit. B12. def. animals for impaired (*p* = 0.17) or non-impaired (*p* = 0.42) limbs. The vit. B12. def. diet animals did take longer to cross the ladder beam compared to the control animals (Figure 7C, *p* = 0.02). 

## 4. Discussion

A vitamin B12 deficiency results in increased risk of stroke and worse stroke outcome in patients [6], and the mechanisms through which this occurs remain unknown. In this study, we investigated the mechanisms through which a vitamin B12 deficiency impacts stroke outcome in aged female mice. After ischemic stroke, vitamin B12-deficient animals had increased levels of total homocysteine in plasma and liver tissues. There were no changes in one-carbon metabolites in plasma, but in liver tissue, the choline levels were increased in the vitamin B12-deficient animals. Vitamin B12-deficient females had larger ischemic damage volume in brain tissue and more apoptosis within the ischemic region. Our study is the first to describe mitochondria metabolite changes in the brain tissue of control and vitamin B12-deficient aged female animals after ischemic stroke. The analysis of the mitochondrial metabolomics in brain tissue showed significant decreases in phenylalanine, malate, tyrosine, aspartate, alanine, valine, isoleucine, and fumarate in response to a vitamin B12 deficiency and not ischemic damage, interestingly. Meanwhile, pathway analysis showed significant, widespread dysfunction in phenylalanine, tyrosine, and tryptophan biosynthesis. Furthermore, in the cecum, we describe changes in methylmalonic acid and creatinine; pathway enrichment analysis showed dysfunction in B12 transport. Motor outcome after ischemic stroke was also measured and showed that vitamin B12-deficient females performed worse on the vertical screen test and had impaired coordination and balance.

We confirmed the dietary vitamin B12 deficiency in animals by measuring the levels of plasma and liver homocysteine. Our metabolite analysis also revealed that the levels of methylmalonic acid were increased in the cecum. Clinically, both increased levels of homocysteine and methylmalonic acid are markers for a vitamin B12 deficiency [38]. Others have used the same vitamin B12-deficient diet used in this study and have demonstrated that it results in a deficiency [24], and our data build upon this literature. Unfortunately, we were unable to measure the levels of vitamin B12 in our animals because we used tissues for other measurements, so we were not able to correlate the levels of vitamin B12 to stroke outcome; this is a future goal of our research. 

The mice used in the present study are defined as aged animals modeling aspects of older humans [39], making this a valid preclinical model. Our data confirm the clinical observations that a vitamin B12 deficiency results in worse ischemic stroke outcome in aged females [5,6]. The data from this study provide insight into how the brain and cecum tissue of vitamin B12-deficient aged females respond to ischemic stroke. We have shown more damage in the brain tissue after ischemic stroke and increased levels of neuronal apoptosis within the damaged region. Our metabolite measurements in healthy and ischemic brain tissue revealed that the metabolites of the tricarboxylic acid (TCA) cycle (malate and fumarate) are reduced in vitamin B12-deficient animals. The TCA cycle has been implicated in the response to hypoxia [40]. The induction of stress such as ischemic stroke may push more neurons to die faster. Our data also report reduced levels of creatine in the cecum of vitamin B12-deficient mice, which has been reported to delay neurodegeneration [41]. Reduced levels of creatine in the cecum may be playing a role in the increased neurodegeneration we observe in the brain tissue of vitamin B12-deficient animals after ischemic stroke. 

Vitamin B12 plays several roles within the cell: it is involved in the methylation of homocysteine and the generation of *S*-adenosylmethionine (SAM), a global methyl donor. Additionally, in the mitochondria, vitamin B12 is involved in the regulation of mitochondrial metabolism. Recent data suggest that vitamin B12 plays an important antioxidant role in the body and, when levels are reduced, there is more oxidation present [42,43]. The increased levels of oxidation lead to the activation of nuclear factor–erythroid factor 2-related factor (Nrf-2) [43], an important transcription factor involved in the cellular response to oxidative stress. Ischemic stroke also results in oxidative stress and increased levels of Nrf-2 [44]. In our previous study, we showed that supplementation with B vitamins, including vitamin B12, results in increased levels of Nrf-2 and superoxide dismutase 2 (SOD2) [19]. Another study has described that vitamin B12 can act as a scavenger for reactive oxygen species (ROS) after renal ischemia and reperfusion, as well as reduce inflammation [45]. Possibly through its antioxidant role, the dietary supplementation of vitamin B12 after stroke has been shown to provide benefits in preclinical models of stroke [19,46], as well as in clinical studies [47,48]. It is important to note that the supplementation of vitamin B12 should be carried out with care and renal function needs to be measured [6,49].

There were limitations to our study. The first is the exclusive use of female mice. There is a bias in preclinical studies that use mostly male mice [8,9], which means clinical pharmaceutical findings favor better outcomes in males [10,11]. However, the impact of vitamin B12 deficiency on stroke outcome on males does need to be investigated, and clinical data have shown that males are more susceptible to vitamin B12 deficiencies [50]. Furthermore, in our study, we measured the levels of total homocysteine in plasma and liver, as well as methylmalonic acid in cecum tissue, both of which are markers of a dietary deficiency of vitamin B12 [38]. The data in the present study could be enhanced if the levels of vitamin B12 also need to be measured in tissue. 

## 5. Conclusions

In conclusion, our stroke outcome data from this study corroborates the current clinical data, specifically, that a vitamin B12 deficiency results in worse stroke outcome. The driving force of this reduced outcome may be changes in mitochondrial metabolism and aminoacyl tRNA biosynthesis, leading to more neurodegeneration in brain tissue and impaired motor function after ischemic stroke. There are sex differences in ischemic stroke in preclinical and clinical studies, as well as in the responses to deficiencies in vitamin B12. Future work could investigate the mechanisms in male animals and whether dietary supplementation with vitamin B12 in stroke animals ameliorates these changes in both sexes. The implications of this work suggest that a vitamin B12 deficiency is detrimental for ischemic stroke outcome and may be an effective target for therapeutics in stroke patients. 

## Figures and Tables

**Table 1:** One-carbon metabolite concentrations in plasma of control and vitamin B12 deficient diet females ^1^.

Concentration of Metabolites µM	CD	Vit. B12 Def.	Diet
Homocysteine	8.25 ± 0.41	14.2 ± 1.15	*p* = 0.0008
*S*-adenosylmethionine	0.34 ± 0.027	0.41 ± 0.059	*p* = 0.29
S-adenosylhomocysteine	0.10 ± 0.017	0.16 ± 0.043	*p* = 0.28
Methionine	85.0 ± 4.33	90.9 ± 7.41	*p* = 0.53
Cystathionine	1.01 ± 88.4	0.95 ± 0.072	*p* = 0.62
Betaine	51.1 ± 4.11	49.8 ± 4.98	*p* = 0.85
Choline	P = 24.6 ± 2.25	29.0 ± 6.01	*p* = 0.55

^1^ Values are means ± SEMs. Abbreviations: CD, control diet; vit. B12 def, dietary vitamin B12 deficiency. Unpaired *t*-test, diet effect.

**Table 2:** One-carbon metabolite concentrations in liver tissue of control and vitamin B12 deficient diet females ^1^.

Concentration of Metabolites µmol/g	CD	Vit. B12 Def.	Diet
Homocysteine	6.39 ± 0.34	8.98 ± 0.40	*p* = 0.001
*S*-adenosylmethionine	547 ± 94.1	797 ± 213	*p* = 0.37
S-adenosylhomocysteine	425 ± 21.7	454 ± 37.1	*p* = 0.53
Methionine	5033 ± 442	4746 ± 362	*p* = 0.62
Cystathionine	256 ± 20.5	249 ± 24.9	*p* = 0.85
Betaine	123 ± 11.2	117 ± 12.8	*p* = 0.73
Choline	257 ± 35.2	373 ± 21.6	*p* = 0.02

^1^ Values are means ± SEMs. Abbreviations: CD, control diet; vit. B12 def, dietary vitamin B12 deficiency.

## Data Availability

Not applicable.

## Supplementary Materials

The following supporting information can be downloaded at: https://www.mdpi.com/article/10.3390/nu14142960/s1. Figure S1: Two-factor heatmap of 34 brain mitochondria metabolites by group/lesion. Figure S2: Correlation and clustering analysis of brain mitochondria metabolites. Figure S3: OPLS-DA and permutation testing with 34 brain mitochondria metabolites. Figure S4: PCA scores plot between study groups using eight significant metabolites. Figure S5: Univariate ROC analysis of phenylalanine and tyrosine for B12 deficiency. Dataset S1: Summary of data.
