# Supplementary material for: Ischemic Stroke and Dietary Vitamin B12 Deficiency in Old-Aged Females: Impaired Motor Function, Increased Ischemic Damage Size, and Changed Metabolite Profiles in Brain and Cecum Tissue
Source: Nutrients. 2022 Jul 19;14(14):2960. doi: 10.3390/nu14142960 (PMC9318046; doi:10.3390/nu14142960)
Supplement: Supplementary file 1 [file nutrients-14-02960-s001.zip › nutrients-1783416-supplementary.pdf]

Supplementary Information

Reduced stroke outcome in old-aged female mice maintained on a dietary vitamin B12 deficiency

Joshua Poole BS[1,2*], Paniz Jasbi MS[3*], Agnes S. Pascual MS[2], Sean North BS[1,2], Neha Kwatra BSc[2,4], Volkmar Weissig PhD[2,5], Haiwei Gu PhD[3,6,7], Teodoro Bottiglieri PhD[8], Nafisa M. Jadavji PhD[2,9,10]

*Equal contributions to the manuscript

[1] College of Osteopathic Medicine, Midwestern University, Glendale, AZ, US

[2] Biomedical Sciences Program, College of Graduate Studies, Midwestern University, Glendale, AZ, US

[3] College of Health Solutions, Arizona State University, Phoenix, AZ, US

[4] College of Dental Medicine Arizona, Midwestern University, Glendale, AZ, US

[5] Department of Pharmaceutical Sciences, College of Graduate Students, Midwestern University, Glendale, AZ, US

[6] Department of Environmental Health Sciences, the Robert Stempel College of Public Health and Social Work, Florida International University, Miami, FL, US

[7] Center for Translational Science, Cellular Biology and Pharmacology Department, the Herbert Wertheim College of Medicine, Florida International University, Port St. Lucie, FL, US

[8] Center of Metabolomics, Institute of Metabolic Disease, Baylor Scott & White Research Institute, Dallas, TX, USA

[9] College of Veterinary Medicine, Midwestern University, Glendale, AZ, US

[10] Department of Neuroscience, Carleton University, Ottawa, Canada

Page S-3, Figure S1 Two-factor heatmap showing normalized relative abundance of 34 captured metabolites of brain mitochondria by group (control vs. vitamin B12 deficient) and lesion status (lesion vs. non-lesion).

Page S-4, Figure S2 Pearson's correlation and clustering heatmap between 34 detected study metabolites from bran mitochondria. Correlation and significance cutoffs were set to $r \geq |0.5|$ and $p < 0.05$, respectively.

Page S-5, Figure S3 Orthogonal partial least squares-discriminanat analysis (OPLS-DA) performed with 34 captured metabolites of the brain mitochondria. (A) OPLS-DA scores plot between control and vitamin B12 deficient groups (Q^2 = 0.435, R^2 = 0.815). (B) Permutation testing with 100 iterations (perm. $p < 0.05$).

Page S-6, Figure S4 Unsupervised principal component analysis (PCA) performed with the subset of eight significant metabolites of the brain mitochondria. PC1 and PC2 explain more than 83% of total variance.

Page S-7, Figure S5 Receiver operating characteristic (ROC) analysis of vitamin B12 deficiency in brain mitochondria. (A) Univariate area under curve (AUC) of phenylalanine (AUC = 0.988, 95% CI: 0.9-1.0, sensitivity = 0.9, specificity = 1.0). (B) Standard box plot of normalized phenylalanine measurements showing optimal cutoff (red line) and group means (yellow diamonds). (C) Univariate AUC of tyrosine (AUC = 0.925, 95% CI: 0.75-1.0, sensitivity = 0.8, specificity = 0.9). (D) Standard box plot of normalized tyrosine measurements showing optimal cutoff (red line) and group means (yellow diamonds).

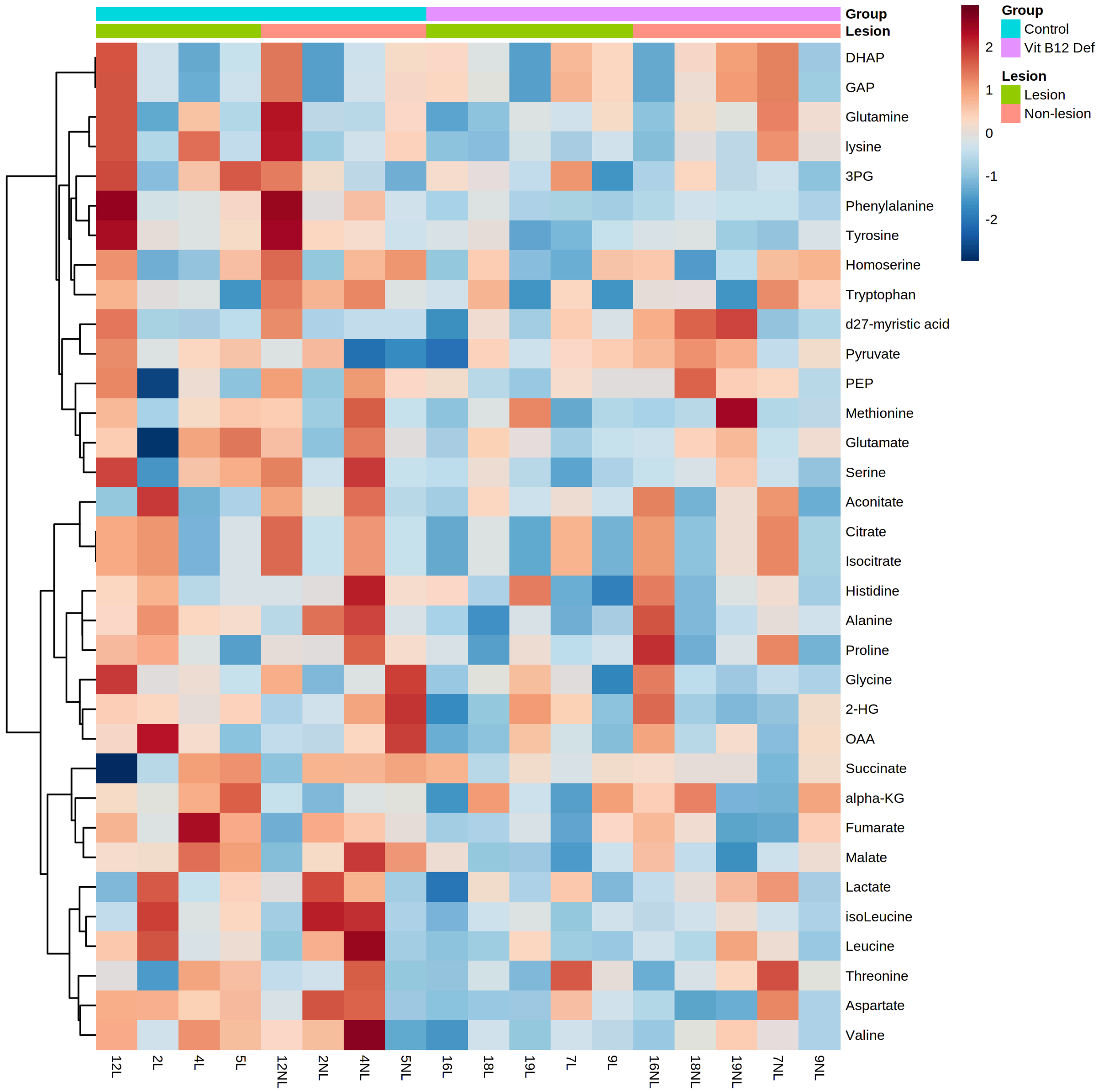

Figure S1 Two-factor heatmap showing normalized relative abundance of 34 captured metabolites of brain mitochondria by group (control vs. vitamin B12 deficient) and lesion status (lesion vs. non-lesion).

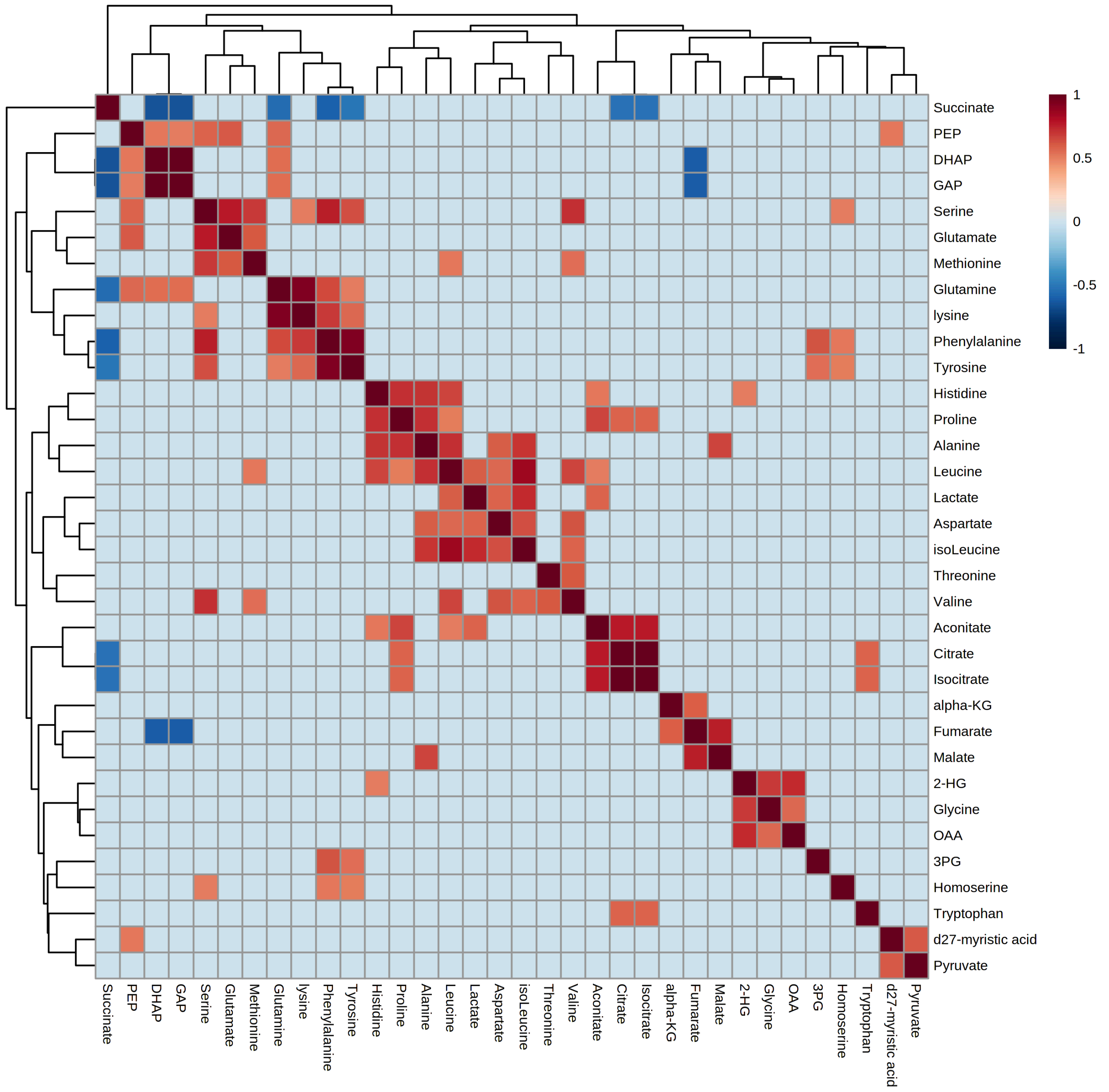

Figure S2 Pearson's correlation and clustering heatmap between 34 detected study metabolites from bran mitochondria. Correlation and significance cutoffs were set to $r \geq |0.5|$ and $p < 0.05$, respectively.

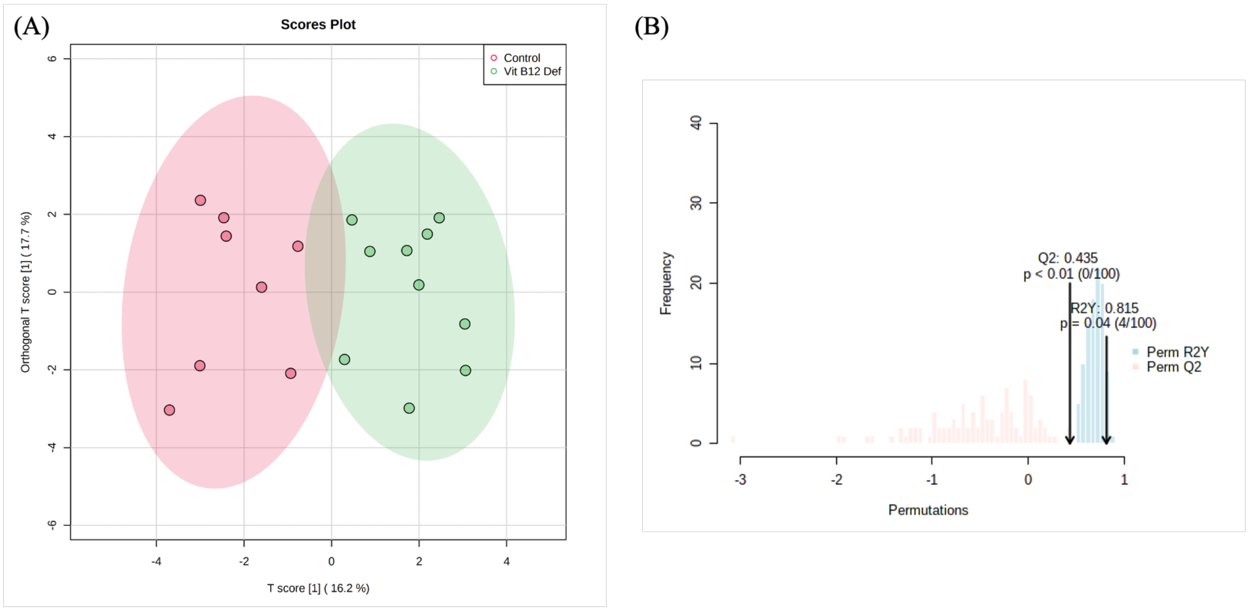

Figure S3 Orthogonal partial least squares-discriminanat analysis (OPLS-DA) performed with 34 captured metabolites of the brain mitochondria. (A) OPLS-DA scores plot between control and vitamin B12 deficient groups ($Q^2 = 0.435$, $R^2 = 0.815$). (B) Permutation testing with 100 iterations (perm. $p < 0.05$).

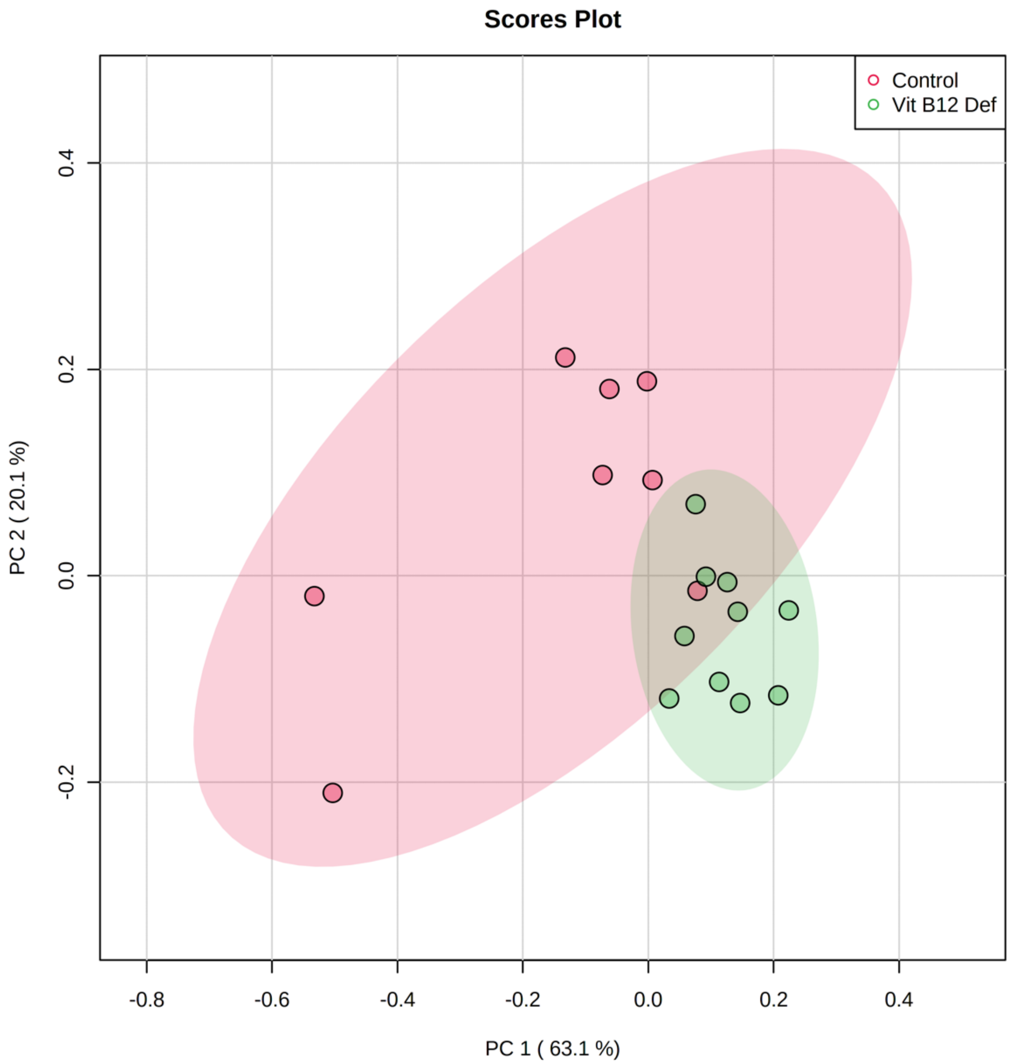

Figure S4 Unsupervised principal component analysis (PCA) performed with the subset of eight significant metabolites of the brain mitochondria. PC1 and PC2 explain more than 83% of total variance.

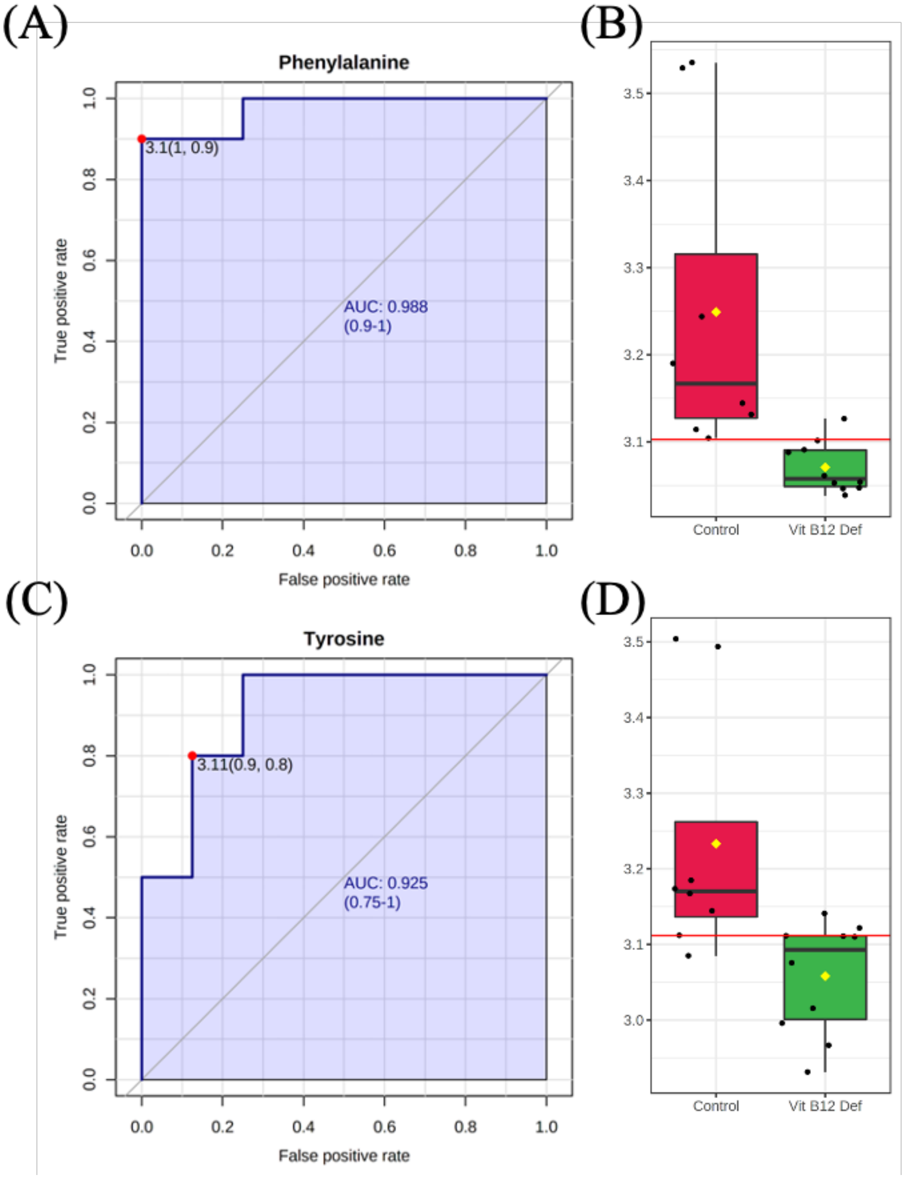

Figure S5 Receiver operating characteristic (ROC) analysis of vitamin B12 deficiency in brain mitochondria. (A) Univariate area under curve (AUC) of phenylalanine (AUC = 0.988, 95% CI: 0.9-1.0, sensitivity = 0.9, specificity = 1.0). (B) Standard box plot of normalized phenylalanine measurements showing optimal cutoff (red line) and group means (yellow diamonds). (C) Univariate AUC of tyrosine (AUC = 0.925, 95% CI: 0.75-1.0, sensitivity = 0.8, specificity = 0.9). (D) Standard box plot of normalized tyrosine measurements showing optimal cutoff (red line) and group means (yellow diamonds).
